# Characterization of an Ecdysteroid-Regulated 16 kDa Protein Gene in Chinese Oak Silkworm, *Antheraea pernyi* (Lepidoptera: Saturniidae)

**DOI:** 10.1093/jisesa/ieaa033

**Published:** 2020-05-12

**Authors:** Miao-Miao Chen, Liang Zhong, Chun-Shan Zhao, Feng-Cheng Wang, Wan-Jie Ji, Bo Zhang, Shu-Yu Liu, Yan-Qun Liu, Xi-Sheng Li

**Affiliations:** 1 Research Group of Silkworm Breeding, Sericultural Institute of Liaoning Province, Fengcheng, China; 2 Department of Sericulture, College of Bioscience and Biotechnology, Shenyang Agricultural University, Shenyang, China

**Keywords:** *Antheraea pernyi*, ecdysteroid-regulated 16 kDa protein (ESR16), diapause-related gene, 20-hydroxyecdysone

## Abstract

A large number of ecdysteroid-regulated 16 kDa proteins (ESR16s) of insects have been isolated and annotated in GenBank; however, knowledge on insect ESR16s remain limited. In the present study, we characterized an ecdysteroid-regulated 16 kDa protein gene isolated in Chinese oak silkworm, *Antheraea pernyi* Guérin-Méneville (‘*ApESR16*’ in the following), an important silk-producing and edible insect. The obtained cDNA sequence of *ApESR16* is 1,049 bp, harboring an open reading frame of 441 bp that encodes a polypeptide of 146 amino acids. CD-search revealed that ApESR16 contains the putative cholesterol/lipid binding sites on conserved domain Npc2_like (Niemann–Pick type C-2) belonging to the MD-2-related lipid-recognition superfamily. Sequence comparison revealed that ApESR16 exhibits 51–57% identity to ESR16s of lepidopteran insects, 36–41% identity to ESR16 or NPC2a of nonlepidopteran insects, and 28–32% identity to NPC2a of vertebrates, indicating a high sequence divergence during the evolution of animals. Phylogenetic analysis found that the used sequences were divided into two groups corresponding to vertebrates and invertebrates, and the used insect sequences were also well clustered according to their families. The *A. pernyi ESR16* mRNA is expressed during all four developmental stages and in all tested tissues. Injection of 20-hydroxyecdysone (20-E) into *A. pernyi* diapausing pupae triggering diapause termination induced upregulation of *ESR16* mRNA compared to the diapausing pupae, with the highest expression level at day 2 in the ovaries but day 12 in the fat body. Our results suggested that *ApESR16* might be a diapause-related gene and plays a vital role in the pupal diapause of *A. pernyi*.

Ecdysteroids are arthropod steroid hormones that control development and reproduction. Their main representative is 20-hydroxyecdysone (20-E) synthesized from cholesterol (Lafont et al. 2012). During the molting process, changes in the levels of ecdysteroids can trigger the expression of a new set of genes (Bollenbacher et al. 1981, Mészáros and Morton 1996). 20-Hydroxyecdysone can trigger pupal diapause termination of insects, including the focused Chinese oak silkworm, *Antheraea pernyi* Guérin-Méneville (Denlinger 2008; Liu et al. 2015).

In insects, the ecdysteroid-regulated 16 kDa protein (ESR16) was for the first time identified in the tobacco hornworm, *Manduca sexta* L., and its expression was negatively regulated by the ecdysteroids (Mészáros and Morton 1996). Northern blot analysis has shown that the transcript of *ESRl6* of *M. sexta* can be detected in nervous tissue, muscle, and trachea isolated from individuals 4 h before, but not 24 h before pupal ecdysis. The gene encodes a secreted protein with 35% identity to Niemann–Pick type C-2a (NPC2a), a human epididymal-specific gene that encodes epididymal secretory protein E1 (HE1; Mészáros and Morton 1996, Inohara and Nuñez 2002). Subsequently, research on *Helicoverpa armigera* Hubner (Lepidoptera: Noctuidae) has suggested that ESR16 might be a response protein to 20-E and involved in pupal diapause (Zhu et al. 2010). A recent study has also found that, in *Eriocheir sinensis* Milne Edwards, ESR16 can coordinate growth and maturation during larval development and may be involved in the molting and metamorphosis genetically controlled through endocrine systems (Li et al. 2015).

So far, knowledge on insect ESR16s remain limited, although a large number of ESR16s have been predicted and annotated in GenBank by automated computational analyses. In the present study, an ESR16 gene was isolated from a full-length pupal cDNA library of *A. pernyi* (Li et al. 2009). This insect, originally domesticated in China around the 16th century, is one of the most well-known economic insects used for silk production (Peigler 2012). The larvae, pupae, and moths of this species have also been considered as a source of insect food (Liu et al. 2010). The pupae contain most of the amino acids needed by the human, thus are considered as a newly available high-quality protein food for human consumption (Zhou and Han 2006). To understand the evolutionary relationship, ESR16s and the homologs from insects and noninsect animals were collected and compared. We also examined the expression pattern at four developmental stages and in different tissues of the fifth instar. Finally, we examined the mRNA expression change after injecting 20-E into the diapausing pupae. The results presented here would provide basic information for further functional analysis of ESR16s of insects.

## Materials and Methods

### Insects and Tissues

The larvae of a bivoltine strain *Shenhuang No. 1* of *A. pernyi* used in this study were reared on oak trees (*Quercus wutaishanica* Mayr) in the field. The larvae at day 10 of the fifth instar were used to dissect the various tissues, including hemocytes, fat body, midgut, silk glands, body wall, Malpighian tubules, spermaries (male), ovaries (female), brain, and muscle. The day 10 of the fifth-instar larvae represents the two-thirds duration, and the larvae of the day 10 also are in feeding state. The dissected tissues were immediately frozen in liquid nitrogen and then stored at −80°C until use. Whole moths, pupae, eggs at day 5, and fifth-instar larvae were also collected.

To examine the effect of 20-E on the mRNA expression level of *ApESR16*, we injected 20 µg of 20-E per individual (Tokyo Chemical Industry Co. Ltd., Japan) dissolved in 10% ethanol into the diapausing pupae of *A. pernyi*, and 10% ethanol served as the control. This dose can trigger almost all of the diapausing pupae to initiate the development of *A. pernyi* (Liu et al. 2015). Four microliters of 20-E (5 µg/µl) was injected into the diapausing pupae by abdominal internode membrane using microinjector. The diapause state was determined by examining the pigment-free region of cuticle above the brain (the cuticular window) according to the color change (Liu et al. 2015). All treated pupae and the control were then kept at 25°C and humidity at 70%, under a short-day photoperiod (9:15 [L:D] h). The diapausing pupae of *A. pernyi* would remain in diapause during short-day conditions. The fat body and ovaries of female pupae were dissected every 2 d. Ten individuals were observed for each sampling points.

### Total RNA Isolation and First-Strand cDNA Synthesis

Total RNA was extracted using RNAprep Pure Tissue Kit (TIANGEN Biotech, Beijing, China). The potential genomic DNA was removed by DNase I. The RNA integrity was analyzed by 1.5% (w/v) agarose gel electrophoresis. The RNA purity and quantity were assessed by the ratio of OD_260_/OD_280_ with ultraviolet spectrophotometer. Total RNA of 2 µg was used to generate the first-strand cDNA with TIANScript RT Kit (TIANGEN Biotech) following the manufacturer’s instructions.

### Isolation of *A. pernyi ESR16* cDNA, Sequence Analysis, and Phylogenetic Inference

In our previous study, a pupal full-length cDNA library of *A. pernyi* has been constructed with the Creator SMART cDNA Library Construction kit (Clontech; Li et al. 2009). By random expressed sequence tag (EST) sequencing at the 5′-end on an ABI 3730 Genetic Analyzer (Applied Biosystems) of this full-length cDNA library, a homolog encoding putative ESR16 was isolated. The plasmid carrying the putative full-length *ApESR16* cDNA was then picked out to obtain the remaining 3′-end cDNA fragment by primer walking. ApESR16 was used as input query against Blastp to retrieve the homologs sequences in GenBank. CD-search was used to identify the conserved domain (Marchler-Bauer et al. 2017). Signal peptide prediction was conducted on SignalP-5.0 Server (http://www.cbs.dtu.dk/services/SignalP/) (Almagro Armenteros et al. 2019). ClustalX software was used to align the amino acid sequences (Thompson et al. 1997). Phylogenetic relationship was reconstructed by MEGA 6.0 (Tamura et al. 2013) under the maximum likelihood method, and the bootstrap test was performed with 1,000 replications to test the statistical significance of the nodes. Substitution model selection was performed based on the lowest BIC scores (Bayesian information criterion), and the WAG+G model was selected.

### Reverse Transcription–Polymerase Chain Reaction and Quantitative Analysis

Reverse transcription–polymerase chain reaction (RT–PCR) was used to verify the complete open reading frame (ORF) sequence and investigate the expression levels of *ApESR16* across tissue types of fifth-instar larvae and four developmental stages. The primer pair ORF-F (5′-GGTCG CTCGG AGACG AACAG-3′) and ORF-R (5′-ACTGA TTTTG ATTTT CTTTT ACGC-3′) was used to verify the complete ORF sequence. The gene-specific primer pair LYQ203 (5′-TATCG CAATA CTCCT GGTCG-3′) and LYQ204 (5′-TGAGT TCCCA TTTGA CATCC-3′) was used to detect the expression pattern of *ApESR16*, which would generate a 368 bp fragment. The eukaryotic translation initiation factor 4A gene (*eIF-4A*) served as the internal control (Wang et al. 2008), with the specific primer pair eIF-4A-F (5′-TCCAT CGCTC AGGCT GTTAT-3′) and eIF-4A-R (5′-TCAGA TGAGG TTGGC CACAT CAC-3′), which would amplify a 340 bp fragment (KC481238, Chen et al. 2014). RT–PCR was carried out in a reaction volume of 25 µl, containing 0.5 µl of cDNA sample, each primer (final concentration: 100 nmol/l), dNTP (final concentration: 200 µmol/l), 2.5 µl of 10× buffer (with MgCl_2_), and 1 U Taq DNA polymerase (TIANGEN Biotech). The amplification cycling parameter was initial at 95°C for 3 min, followed by 27 cycles of 1 min at 95°C, 30 s at 55°C, 30 s at 72°C, and a final extension at 72°C for 10 min. The amplification products were analyzed on 1.5% agarose gels stained with ethidium bromide.

Real-time quantitive RT–PCR (qRT–PCR) was also used to determine the mRNA expression levels of *ApESR16*. Primers for qRT–PCR were designed by Beacon Designer 7.7 software (Premier Biosoft International, Palo Alto, CA). The gene-specific primer pairs, RT9 (5′-GTGTA GTGAG GACGC TAA-3′) and RT10 (5′-TCTGT GGTTA TGCTG GAA-3′) for *ApESR16* and RT1 (5′-TCCTC TCGTG TGCTT ATC-3′) and RT2 (5′-CCACC TCTTC CGATT CTAT-3′) for *eIF4A*, were used. qRT–PCR was done in a reaction volume of 10 µl with 2× SYBR solution (TIANGEN Biotech) and under parameters that contain an initial at 95°C for 2 min, followed by 40 cycles (95°C for 15 s, 60°C for 30 s, and 68°C for 30 s), and a final stage of 60–95°C to determine melting curves of amplified products. The 2^−ΔΔCt^ method was used to calculate the relative changes of gene expression (Livak and Schmittgen 2001). Six individuals were used for each sampling time. To eliminate large individual difference, total RNA was extracted individually, quantified, and mixed equally. For each sampling time point, three technical replicates were performed only to check the method’s error and to get the average *C*_t_ value. Three independent biological replicates for each sampling point were performed. A two-tailed Student’s test was used to determine the statistical difference between groups, and *P* < 0.01 was considered to be significant.

## Results

### Identification of *A. pernyi* ESR16 Gene

**Fig. 1.**
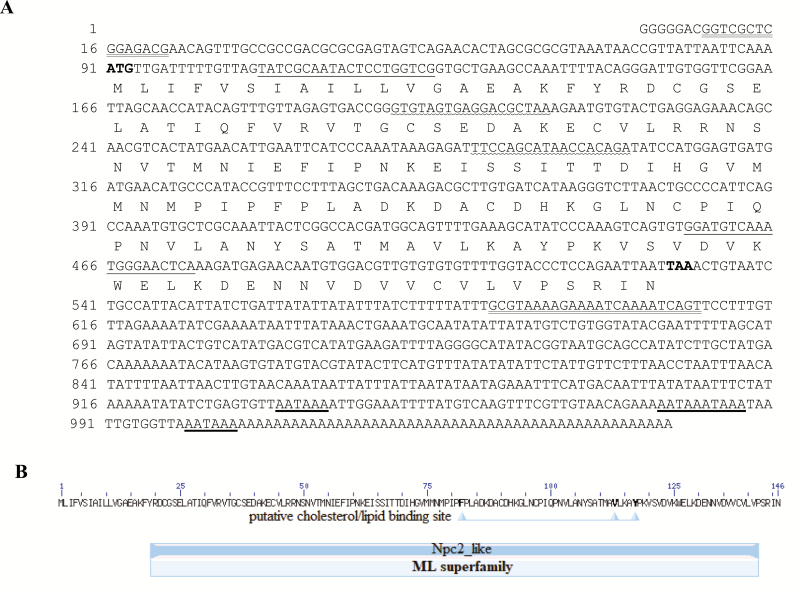
The complete nucleotide, deduced amino acid sequence (A) and putative conserved domain (B) of *Antheraea pernyi* ESR16. The amino acid residues are represented by one-letter symbol. The initiation codon ATG is bolded, and the termination codon TAA is bolded and marked with an asterisk (*). The polyadenylation signals AATAAA are thick underlined. The gene-specific primer sequences used in the (q)RT–PCR experiment are underlined. The putative conserved domain of Npc2_like (Niemann–Pick type C-2) in ApESR16 was determined by CD-search.

The full-length cDNA of *ApESR16* was isolated from a pupal full-length cDNA library constructed with the SMART technique. The obtained 1,049 bp cDNA sequence comprises a 5′ untranslated region (UTR) of 90 bp, a partial 3′ UTR of 523 bp, and an ORF of 441 bp that encodes a polypeptide of 146 amino acids, with a canonical polyadenylation signal sequence AATAAA (Fig. 1; GenBank MG020560). We also observed other three polyadenylation signal sequence AATAAA in its 3′-UTR sequence. The complete ORF sequence was also verified by RT–PCR amplification and sequencing. The deduced amino acid sequence has a predicted molecular weight of 16.15 kDa and an isoelectric point of 5.33. Signal peptide prediction showed that ApESR16 contains a cleavage site between positions 16 and 17 (data not shown), indicating that it is a secretory protein, like SER16, identified in *M. sexta*. CD-search revealed that ApESR16 is a Npc2_like domain-containing protein (domain architecture ID 10097044) and shares several conserved features/sites of MD-2-related lipid-recognition (ML) family that was implicated in lipid recognition or metabolism, such as putative three cholesterol/lipid-binding site and six cysteine residues (Inohara and Nuñez 2002, Shi et al. 2012).

### Homologous Alignment

**Fig. 2.**
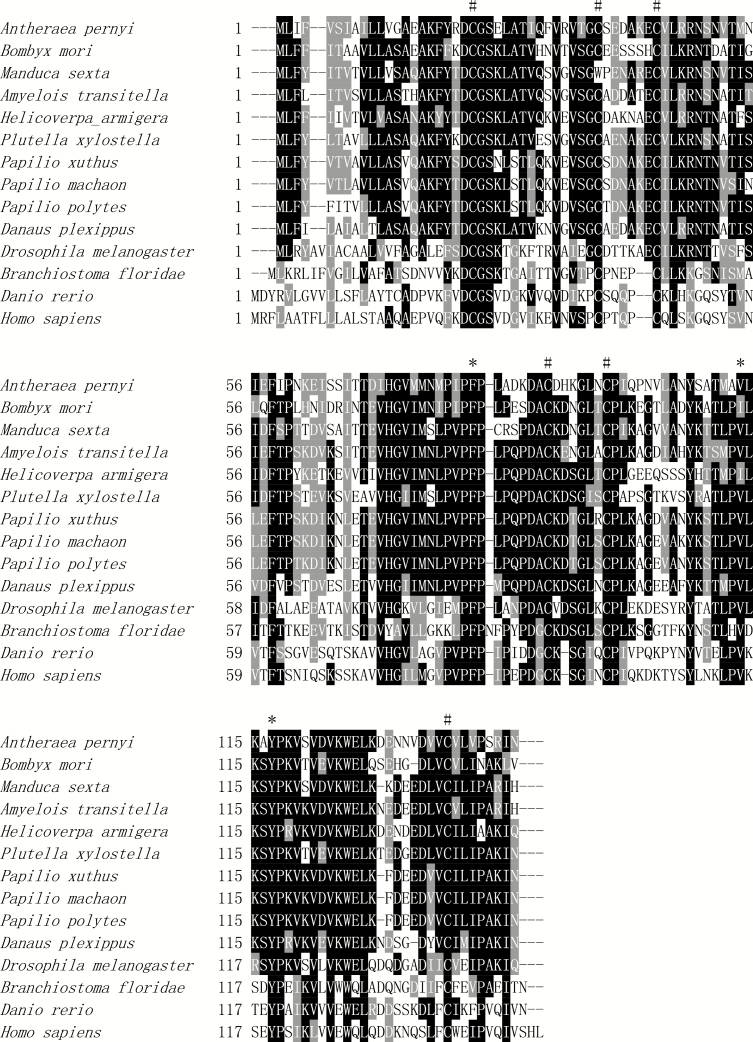
Sequence alignment of ESR16s and homologs of lepidopteran insects, nonlepidopteran insects, and noninsect animals. The sign asterisk (*) shows the position of the putative cholesterol/lipid binding site. The sign asterisk (#) shows the position of conserved cysteine residues. The alignment was generated using ClustalX, together with the Boxshade server.

By blast searching against GenBank database, there are nine sequences of ESR16 available in lepidopteran species, including five moths, *Bombyx mori* L. (NP_001093080), *M. sexta* (Q25481), *Amyelois transitella* (Walker) (XP_013192310), *Plutella xylostella* L. (XP_011568846), *H. armigera* (XP_021184351), and four butterflies, *Papilio xuthus* L. (NP_001299692), *P. machaon* L. (XP_014364392), *P. polytes* L. (XP_013136334), and *Danaus plexippus* L. (EHJ65928). However, only two ESR16s from *M. sexta* and *H. armigera* were partially characterized at the mRNA or protein level (Mészáros and Morton 1996, Zhu et al. 2010). Sequence comparison revealed that ApESR16 shares the highest identity (57%) with the ESR16s from *M. sexta* and *A. transitella*, and only 51% identity with the ESR16 from *B. mori*. Note that the identities between *A. pernyi* and butterflies used are larger than that between *A. pernyi* and *B. mori*, both of them belong to silkworm (Fig. 2). A large number of homologs including ESR16, NPC2a, and HE1 are also retrieved from nonlepidopteran insects and noninsect animals. In total, 33 representative ESR16s and homologs were used to calculate the sequence identity, including invertebrate (25) and vertebrate (8). Sequence comparison indicated that ApESR16 shows 36–41% identity to ESR16/NPC2a of nonlepidopteran insects, 34% identity to a hypothetical protein of *Branchiostoma floridae* Hubbs (XP_002610716), and 28–32% identity to NPC2a of vertebrates. All these sequences share the conserved domain of NPC2, including three cholesterol/lipid-binding sites and six cysteine residues, although they exhibit a low degree of identity (Fig. 2).

### Phylogenetic Analysis

**Fig. 3.**
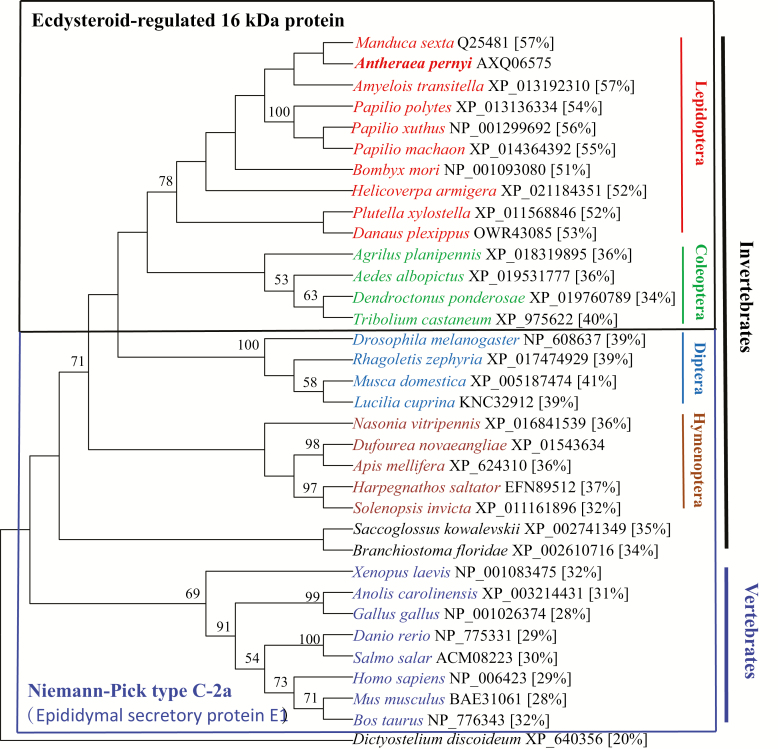
Maximum likelihood tree based on the amino acid sequence comparisons of ESR16s and homologs of animals. The topology was tested using bootstrap analyses (1,000 replicates). Public database accession numbers of proteins are shown following the names of animals. Identity (%) in parentheses following accession number was calculated by pairwise alignment of protein sequence of ApESR16 with indicated homologs.

In total, 33 representative ESR16s and homologs were used to infer the phylogenetic relationship. The sequence from a hypothetical protein (XP_640356) of *Dictyostelium discoideum* belonging to ML superfamily and exhibiting a 20% identity to ApESR16 served as outgroup. In the phylogenetic tree (Fig. 3), all used protein sequences were well divided into two groups, invertebrates (insects and Lancelet) and vertebrates. The amphioxus *B. floridae* was placed between insects and vertebrates. The sequences from vertebrates were divided into four subgroups corresponding to amphibious, birds, fishes, and mammals. The used sequences of insects were also well clustered into four subgroups corresponding to Lepidoptera, Coleoptera, Diptera, and Hymenoptera. The phylogenetic relationships of ESR16s and homologs sequences followed the classical evolutionary trends. The exception was *Aedes albopictus* (Skuse) that morphologically belongs to Diptera was placed into the subgroup of Coleptera. Note that neither protein sequences (Fig. 3) nor nucleotide sequences (data not shown) could separate six moths from four butterflies.

### Developmental and Tissue Expression Pattern

**Fig. 4.**
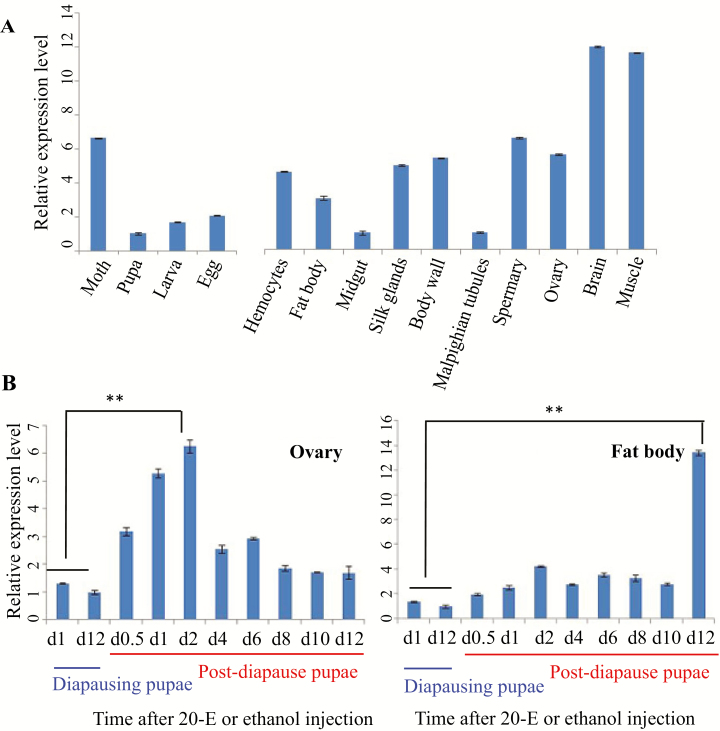
Expression of *Antheraea pernyi ESR16*. (A) qRT–PCR results for four developmental stages and larval tissues. (B) qRT–PCR results for the ovaries and fat body in diapausing and postdiapause pupae. Postdiapause pupae were activated by 20-E injection. Six individuals were used for each sampling time, and the total RNA was extracted individually, quantified, and mixed equally to eliminate large individual difference. Three biological replicates for each sampling point were performed, and they exhibited similar expression patterns. The mean values and the error bars as indicated were calculated from three technical replicates, representing only one pooled biological sample.

Both RT–PCR and qRT–PCR were used to detect the expression levels of *ApESR16* across tissue types of fifth-instar larvae and developmental stages under nonstressed conditions (Fig. 4A and data not shown). The positive RT–PCR product was sequenced to confirm that they came from *ApESR16* sequence. The *ApESR16* mRNA was found throughout four developmental stages (egg, larva, pupa, and moth) with variation among stages, and in all the tissues tested (hemocytes, fat body, midgut, silk glands, body wall, Malpighian tubules, spermaries, ovaries, brain, and muscle), with low expression in midgut and Malpighian tubules.

### *ESR16* mRNA Expression Changes after 20-E Injection

Morphologically, under the conditions of this study, no obvious changes in the fat body and ovaries could be observed between day 1 and day 12 of the control group that remained in the diapause state by examining the cuticular window, while marked changes could be seen in the treatment pupae. At day 14 after treatment, the cuticular window of the 20-E-treated pupae has lost its transparency and becomes milk white, indicating that they have been postdiapause state. The tough and net-like fat body at day 1 has become soften and curd-like at day 14 after 20-E injection. The ovaries become bigger gradually in size, and eggs began to take shape after day 4 of 20-E injection. All the treated pupae emerged as adults in 25–30 d after 20-E injection under short-day photoperiod, whereas the control pupae remained in the diapause state. Once diapause was terminated with 20-E, the development timetable for adult development was identical to that observed in the nondiapausing pupae. We found that to sample the brain is very difficult in the pupae day 6 after 20-E treatment, while to sample the fat body and the ovaries is difficult day 14 after 20-E treatment due to tremendous changes. Thus, the fat body and the ovaries of pupae from day 0.5 to day 12 after treatment were collected to investigate the *ESR16* mRNA expression change, since it is easy to sample during this stage.

qRT–PCR was also used to determine the expression changes of *ApESR16* in the fat body and ovaries of the diapausing pupae and postdiapause pupae after 20-E injection (Fig. 4B). We found that no significant expression changes of *ApESR16* were observed between day 1 and day 12 of the control group, but the *ApESR16* expression levels were upregulated in both tissues after 20-E injection compared with the diapause state. In the ovaries, the relative expression level of *ApESR16* was significantly upregulated on day 2 after injection (*P* < 0.01 and fold change = 6.3). In the fat body, however, the upregulation of *ApESR16* reached the highest on day 12 after 20-E injection (*P* < 0.01 and fold change = 13.4).

## Discussion

The present study for the first time characterized insect ESR16 proteins, also named as NPC2a in some insects, under the evolutionary frame. In insects, a large number of ESR16s, arbitrarily named ESR16 or NPC2a in different species, have been predicted. Here, the available insect ESR16s and homologs from insects and noninsect animals were collected to investigate the sequence characteristics, infer the evolutionary relationship and compare the expression pattern. Blastp search revealed that the homologs of ApESR16 are present across the animals including invertebrates and vertebrates, and phylogenetic analysis indicated that their phylogenetic relationships follow the classical evolutionary trends. Sequence comparison revealed that ApESR16 only exhibits 28–57% identity to the homologs from invertebrates and vertebrates, indicating a high sequence divergence throughout the evolution of animals. CD-search indicated all of them possess the putative cholesterol/lipid-binding sites on conserved domain Npc2_like (Niemann–Pick type C-2) and six cysteine residues. In all the ESR16s and the homologs, the first and third cholesterol/lipid binding sites are phenylalanine (F) and tyrosine (Y), and the second site is valine (V) or isoleucine (I). The amino acids change from valine to isoleucine only occurred in several animals. These highly conserved functional sites suggested that they undertake similar function throughout evolution of animals.

The NPC2/HE1 protein is not only first characterized as a major secretory protein in the human epididymis, but also detected in most tissues (Vanier and Millat 2004). The available RNA-seq resources in NCBI also indicated that *Npc2a* was detectable in 27 different human tissues with the highest expression level in lung (Fagerberg et al. 2014). A broad expression pattern of *NPC2a* mRNA has also been observed in the fruit fly, *Drosophila melanogaster* Meigen, a model Diptera insect (Huang et al. 2007). The ecdysteroid-regulated developmental events have also been studied in Lepidoptera insect *M. sexta* (Weeks and Levine 1992; Mészáros and Morton 1996, 1997), and northern blot analysis has revealed that the transcript of *ESRl6* can be detected in nervous tissue, muscle, and trachea (Mészáros and Morton 1996). In another Lepidoptera insect *H. armigera*, *ESR16* mRNA could be detected in all tissues, such as brain, suboesophageal ganglion, thoracic ganglion, abdominal ganglion, midgut, and fat body (Zhu et al. 2010). In the present study, we used the lepidopteran insect *A. pernyi* as the experimental material to investigate the expression pattern of the transcript of *ESRl6* and found that, based on qRT–PCR analysis, *ESR16* mRNA was expressed throughout four developmental stages and in all tested tissues of the fifth-instar larvae, with low expression in midgut and Malpighian tubules. We also in silico investigated the expression pattern of *ESR16* in a Lepidoptera model insect *B. mori*, in which large-scale EST resource and extensive microarray information have been available at NCBI and SilkDB (Duan et al. 2009). Based on the EST resources (Bmo.2070; http://www.ncbi.nlm.nih.gov/UniGene/lbrowse2.cgi), *B. mori ESR16* is observed at four developmental stages and expressed in the eye, antenna, pheromone gland, and maxilla. Based on the microarray database at SilkDB (BGIBMGA008405-PA/sw11619), *B. mori ESR16* mRNA is also present in spermaries, ovaries, brain, body wall, fat body, silk glands, and hemocytes of the fifth-instar larvae, with low expression or no expression in the midgut and Malpighian tubules. The expression pattern of *ESR16* in *B. mori* agrees well with those in *A. pernyi* and *H. armigera*. Taken together, these results indicated that in animals *ESR16* or *Npc2a* would expresse throughout the developmental stages and in all tissues, but with a tissue-specific pattern dependent on the investigated insect species.

A previous study has suggested that ESR16 might be involved in pupal diapause of *H. armigera*, where *ESR16* mRNA of the diapausing pupae was lower than the nondiapausing pupae (Zhu et al. 2010). In *H. armigera* brain, *ESR16* was significantly upregulated in the pupae of diapause termination by injecting 20-E, compared with the diapausing pupae. This case is also true in *A. pernyi* ovaries and fat body, where *ESR16* was significantly upregulated in the postdiapause pupae after injection of 20-E compared with the diapausing pupae. These results presented here suggested that ESR16 might be involved in pupal diapause, at least in lepidopteran insects. More works such as gene-knockdown methods should be done to explore the function of ESR16 during the pupal diapause development of insects.

In insects, the ovaries play a vital role in the reproductive system and ecdysteroidogenesis, in which the cholesterol is richer than most of the other tissues (McKinney 2018), and the ovary development is related to lipid metabolism. The fat body of insects also plays an essential role in energy storage and utilization, in which lipid is the main component (Li et al. 2019). So, this study focused on the two important tissues on the morphological changes and the expression changes of *ESR16* mRNA after 20-E injection that triggers the pupal diapause termination. As for the control pupae, no obvious morphological and *ESR16* mRNA expression changes in the fat body and ovaries between day 1 and day 12 indicated that the pupae also remained the diapause state. However, marked morphological and *ESR16* mRNA expression changes between the control and the treated pupae indicated the diapause of the treated pupae by 20-E was completely terminated. Our data indicated that 20-E injection induced upregulation of *ESR16* mRNA in the ovaries and fat body of *A. pernyi*, but with the highest expression level on day 2 in the ovaries and day 12 in the fat body, respectively. The significance of the expression time difference between the ovaries and the fat body should be addressed in the future. Accordingly, the available data further suggested that ESR16 might be involved in pupal diapause of insects by mediating lipid metabolism.

In mammals, the product of *Npc2a* exhibits intermembrane cholesterol transfer activity and can specifically bind cholesterol (Okamura et al. 1999; Infante et al. 2008). Previous work in *D. melanogaster* have also suggested that NPC2a may play a role in the immune deficiency pathway and functions redundantly with NPC2b in regulating sterol homeostasis and ecdysteroid biosynthesis, probably by controlling the availability of sterol substrate (Shi et al. 2012). Accordingly, the upregulation of *ESR16* mRNA induced by 20-E in *H. armigera* (Zhu et al. 2010) and *A. pernyi* presented in this study makes it reasonable to suppose that ESR16 could bind 20-E and/or has cholesterol transfer activity. However, another possibility should be noted that more evidences need to verify that ESR16 is an ecdysteroid-regulated protein, since Niemann–Pick type proteins identified in insects and other organisms might not be under the regulation of ecdysteroid.
